# Andrographolide alleviates paraquat-induced acute lung injury by activating the Nrf2/HO-1 pathway

**DOI:** 10.22038/IJBMS.2023.68827.15000

**Published:** 2023

**Authors:** Degang Zhang, Fengqin Shen, Shiting Ma, Suting Nan, Yanhong Ma, Lili Ren, Hao Li, Qin Yu

**Affiliations:** 1 Lanzhou University, Lanzhou, Gansu Province, 730000, P.R. China; 2 Health Science Center, Lanzhou University, Lanzhou, Gansu Province, 730000, P.R. China; 3 Respiratory and Critical Care Medicine, The first hospital of Lanzhou University, Lanzhou, Gansu Province, 730000, P.R. China

**Keywords:** Acute lung injury, Andrographolide, Apoptosis, Nrf2, Oxidative stress, Paraquat

## Abstract

**Objective(s):**

Paraquat (PQ), a highly effective and rapidly non-selective herbicide, mainly targets the lungs and causes acute lung injury (ALI). So far, the scarcity of effective drug candidates against PQ-induced ALI remains a big challenge. Andrographolide (Andro), with its anti-inflammatory and antioxidant activities, has been demonstrated to alleviate ALI. Nevertheless, whether Andro could alleviate the PQ-mediated ALI remains unknown. Therefore, this study will explore the effects as well as the possible mechanism of Andro against ALI caused by PQ.

**Materials and Methods:**

C57BL/6J mice were injected with 20 mg/kg PQ intraperitoneally to establish an ALI model. PQ-treated MLE-12 cells were applied to a vitro model. Nuclear factor erythroid like-2 (Nrf2) was knocked out to explore the specific effects of the Nrf2/ Heme oxygenase-1 (OH-1) pathway in the protection of Andro against ALI caused by PQ.

**Results:**

Andro significantly reduced lung damage and the ratio of Wet/Dry (W/D) weight, decreased MDA, IL-6, IL-1β, and TNF-ɑ levels, reversed the decrease of CAT and SOD levels, and inhibited apoptosis caused by PQ. Andro obviously increased the ratio of Bcl-2/Bax while reducing caspase-3 and cleaved caspase-3 levels. Furthermore, Andro dramatically elevated the antioxidant proteins Nrf2, NQO-1, and HO-1 levels compared with the PQ group. This experiment demonstrated that Andro reduced ROS and inhibited apoptosis, induced by PQ in MLE-12 cells, by inducing Nrf2/HO-1 pathway activation.

**Conclusion:**

Andro effectively ameliorates oxidant stress and apoptosis in ALI caused by PQ, possibly through inducing Nrf2/HO-1 pathway activation.

## Introduction

Paraquat (PQ, 1,1-dimethyl-4,4-bipyridinium dichloride) is an effective herbicide widely used in agriculture that is extremely toxic to humans (1, 2). More than 150,000 suicides were reported annually owing to accidental or intentional PQ exposure by 2020 (3). Therefore, poisoning by PQ herbicide remains a major public health issue with high fatality rates and no specific antidote (4-6). Some treatment approaches, including administration of gastric lavage, hemodialysis, immunosuppression with dexamethasone, cyclophosphamide, methylprednisolone, and even lung transplantation, are used for PQ poisoning, however, their effects remain uncertain (7). Thus, exploring novel strategies and treatment methods to reverse PQ-induced poisoning remains challenging. 

PQ is chemically similar to polyamines that are abundant in alveolar types I and II, and Clara cells. They are actively transported by the polyamine system, and tend to accumulate in the lungs (8). In the lungs, PQ may disrupt mitochondrial function, cause free radical overproduction, induce inflammation, and induce apoptosis, ultimately leading to ALI and irreversible pulmonary fibrosis, which are responsible for PQ-induced lung toxicity (9). The pathological changes in PQ-induced ALI are associated with alveoli pneumocyte loss/necrosis, edema hemorrhage, and fibrin deposition within the alveoli. Currently, accumulating evidence supports that oxidant damage, inflammation, and apoptosis are the major processes involved in ALI caused by PQ (3, 10-13). In fact, reactive oxygen species (ROS) are the initial factor and the main mechanism in response to inflammation reactions, oxidant stress, and apoptosis after PQ exposure (14). The principal source of ROS is mitochondrial malfunction. Excessive ROS result in the consumption of SOD and CAT, disrupt cellular proteins and lipids and finally give rise to oxidant stress injury (15). Malondialdehyde (MDA) is the main byproduct of lipid peroxidation and a key indicator of the severity of oxidant stress induced by ROS in PQ-induced lung toxicity (16). Excessive ROS and MDA induce cellular dysfunction by means of impairing the stability of the membrane, altering cell signaling, disturbing the transport mechanisms, activating inflammatory reactions, and promoting apoptosis with PQ exposure (17).

Many recent investigations have found that nuclear factor erythroid like-2 (Nrf2) plays an essential role in oxidative stress prevention by lowering ROS and inhibiting MDA (18, 19). As the most important anti-oxidant transcription factor, Nrf2 is in charge of regulating genes that code for anti-oxidant enzymes in order to eliminate the overproduction of ROS. Generally, Nrf2 binds closely to the Kelch-like ECH-associated protein 1 (Keap 1), promoting Nrf2 ubiquitination by E3 ubiquitin ligase, and consequential degradation through the 26S proteasome (20). However, Nrf2 immediately dissociates from Keap 1 and tends to bind to the anti-oxidant response element (ARE) in the nucleus’ DNA, which promotes phase-II detoxing enzymes including NAD(P)H dehydrogenase (quinone1) (NQO-1), and heme oxygenase-1 (HO-1) to generate a large number of anti-oxidants such as SOD and CAT to sustain oxidation-anti-oxidant balance after ROS stimulus (21, 22). Therefore, the anti-oxidant transcription key factor Nrf2 has a significant effect in withstanding the invasion of ROS invasion and maintaining the oxidation and anti-oxidation balance. 

*A. paniculata* is widely distributed in Southeast Asia, including China, Thailand, and Vietnam, and is well-known for treating a variety of ailments such as upper respiratory infections and flu as a form of traditional medicinal herb (23, 24). Andrographolide (Andro), the main substance derived from *A. paniculata*, belongs to the group of diterpenoid compounds with a wide range of biological effects, including anti-inflammatory and antiviral properties (25, 26). Meanwhile, Andro appears to protect against conditions associated with increased oxidative stress by decreasing ROS production and increasing the generation of anti-oxidants, including SOD and CAT. Furthermore, some studies suggest that Andro promotes Nrf2/HO-1 pathway activation to increase the activity of anti-oxidants in liver injury and subarachnoid hemorrhage (27-29). However, whether Andro protects the lungs against PQ-induced lung toxicity remains to be realized. As a result, the specific effects of Andro on ALI caused by PQ were discussed, by lowering oxidative injury and reducing apoptosis. Furthermore, the Nrf2/HO-1 signaling pathway, as well as its effects on PQ-induced oxidative injury and apoptosis, were also explored.

## Materials and Methods

***Animals***

Specific pathogen-free (SPF) female C57BL/6J mice, aged 4–6 weeks and weighing 18–22 g were obtained from the Lanzhou Veterinary Research Institute of the Chinese Academy of Agricultural Sciences. All mice were kept in a day-night (12 hr-12 hr) to acclimate to the SPF environment with controlled temperature (18–22 °C) and humidity (50–70%) for a week. The Ethics Committee of Lanzhou University Second Hospital authorized the experiments (Approval number: D2020-82).

***ALI models***

The mice were randomized into 5 groups: Control group, Andro (Sigma-Aldrich, St. Louis, MO, USA) alone group, PQ (Sigma-Aldrich, St. Louis, MO, USA) group, a low dose, and a high dose of the Andro group. To establish an ALI model, mice were injected intraperitoneally with PQ at a dose of 20 mg/kg, while the Control and Andro groups received an equal volume of saline. The mice in the low and high-dose Andro groups were respectively administered intragastrically daily for 3 days with Andro at doses of 25 mg/kg and 50 mg/kg after PQ exposure for 1 hr. Furthermore, the Control and PQ groups were administered with an equal volume of saline, and the Andro alone group was treated with an equal volume of Andro at 50 mg/kg by intragastric injection.

***Wet/Dry weight ratio in lungs***

After being exposed to PQ for 3 days, lung tissues on the left side were extracted and weighed to determine the weight. The left lung tissues were then baked for 48 hr at 60 °C to get the weight. Ultimately, the ratio of Wet/Dry weight was determined and collected.

***HE and masson staining***

Lung tissues from the right middle lobe were first stained in 4% paraformaldehyde for 72 hr. Lung tissues were subsequently dehydrated by using a concentration gradient, wax dipping, and embedding in paraffin. Paraffin-encased lung tissues were then cut into slices with a thickness of about 5 μm. Finally, slices were stained with hematoxylin and eosin (H&E) and Masson’s trichrome kits (Solarbio, China). Pathological changes in tissues were observed with a microscope (Olympus BX53+DP74) and recorded by the IrfanView 64 software (version 4.54). The evaluation of lung injuries was then based on a 5-point scale. In short, the pathological changes (alveolar pneumocyte loss or necrosis, edema hemorrhage, and fibrin deposition inside the alveoli) were graded on a 5-point scale: The numbers 0 through 4 represented no or minimal, mild, moderate, severe, and highly serious lesions, respectively. At last, the mean scores of each group were used to assess the severity of pulmonary edema.

***TUNEL staining***

The terminal deoxynucleotidyl transferase dUTP nick-end labeling (TUNEL) staining was carried out in conformance with the manual (Servicebio, China). Apoptotic cell nuclei were dyed red and recorded using the IrfanView 64 software (version 4.54). To determine the apoptotic cell counts, five fields were picked at random under a high-power microscope (200× magnification).

***Immunohistochemical (IHC) assay***

Slices of 5 µm-thick lung tissue were blocked with 5% BSA (Biosharp, China) for 1 hr at room temperature, and incubated with Nrf2 (1:200) antibodies overnight at 4 °C. And then, the lung tissue section was incubated for 1 hr with a goat anti-rabbit IgG secondary antibody (1:1000), followed by 8 min of DAB staining. The results were obtained using an Olympus BX53+DP74 fluorescent microscope and recorded by the IrfanView 64 software (version 4.54).

***Detection of IL-1β, IL-6, TNF-α, MDA, SOD, and CAT***

Lung tissues were weighed and homogenized with RIPA (Beyotime, China) for 30 min before centrifugation at 14000 rpm for 20 min at 4 °C to obtain a supernatant for subsequent assays. Protein concentrations were determined by using the BCA Protein Assay Kit (Beyotime, China). Inflammatory cytokines including Interleukin-1β (IL-1β), Interleukin-6 (IL-6), and Tumor Necrosis Factor-α (TNF-α) were measured using the Enzyme-Linked Immunosorbent Assay (ELISA) kits (Biosharp, China). The kits (Nanjing Jiancheng Bioengineering Institute) were intended to assess MDA, SOD, and CAT levels in the lungs. Absorbance was measured with a Thermo Scientific Mulltiskan FC Apparatus (Thermo Fisher, USA).

***Cells and cell culture***

Mouse alveolar type II cells (MLE-12) were supplied by the American Type Culture Collection (ATCC) (Rockville, MD, USA). In brief, MLE-12 cells were cultured in DMEM/F-12 (Gibco, USA) with 10% fetal bovine serum (FBS) (Cell Box, China) and 1% antibiotics, including 10000 U/ml penicillin and 10 mg/ml streptomycin (Biosharp, China), cultivated in a 5% CO_2_ incubator at 37 °C and then trypsinized for passage (Gibco, USA).

***Assays for cell counting kit (CCK-8)***

Cell viability of PQ and Andro were respectively determined using a CCK-8 Assay (Beyotime, China). First, MLE-12 cells were plated at a density of 1×10^4^/well in 96-well plates for 24 hr. Second, MLE-12 cells were treated separately for 24 hr with different doses of PQ (0, 200, 400, 800, 1200, and 2000 μM) and Andro (0, 5, 10, 25, 100, and 400 μM). Following treatment for 24 hr, each well received 10 μl CCK-8, which was then cultured for 1 hr in a 5% CO_2_ incubator at 37 °C. At last, absorbance at 450 nm was detected by the Thermo Scientific Mulltiskan FC apparatus (Thermo Fisher, USA).

***Cell transfection***

Cells were cultivated for 24 hr before being transfected with lentivirus, as directed by the manufacturer (HANBIO, China). The lentivirus transfection sequence is as follows: the sense strand is 5’-GACUCAAAUCCCACCUUAAdTdT-3’ and the anti-sense strand is 5’-UUAAGGUGGGAUUUGAGUCdTdT-3’. After transfection for 48 hr, the transduced cells were cultivated for 2 days with 4 μg/ml puromycin. The cells were then prepared for further studies.

***ROS assay***

The ROS level was assessed by a fluorescent probe, DCFH-DA. DCFH-DA (Biosharp, China) at a concentration of 5 μM was added to each well, which was diluted in a serum-free medium, followed by incubation for half an hour in an incubator at 37 °C. The images of ROS were captured by an Olympus Fluorescence microscope (Olympus IX 53, Tokyo, Japan).

***Apoptosis detection***

Annexin V-FITC/PI labeling (Biosharp, China) was used to identify apoptosis. In brief, cells were resuspended in 500 μl buffers before dealing with the Annexin V-FITC/PI reagent at 37 °C for 30 min. Ultimately, all samples were subjected to CytoFLEX flow cytometry analysis (Beckman, USA).

***Western blot***

RIPA (Beyotime, China) was used to extract total proteins, and then total protein concentrations were measured using the BCA Protein Assay Kit (Biosharp, China). The SDS-PAGE was used to separate distinct proteins with varied molecular weights, and the samples were then transferred onto a PVDF membrane. After blocking for 1 hr with 5% BSA, the membrane was respectively incubated overnight at 4 °C with primary antibodies, including Nrf2 (Abconol, 1:1000), HO-1 (Abcam, 1:10000), NQO-1 (Abconol, 1:1000), Caspase 3 (Abcam, 1:1000), Bcl-2 (Proteintech, 1:1000), cleaved Caspase 3 (Abcam, 1:1000), Bax (Proteintech, 1:5000), and β-actin (Servicebio, 1:2500). Membranes were incubated with the horseradish peroxidase-conjugated goat anti-rabbit IgG (H+L) (Biosharp, 1:10,000) secondary antibody for 1 hr following three TBST (Servicebio, China) washes. Finally, the proteins were visualized by ECL (Biosharp, China) after three washes with TBST. Western blots were finally quantified by Image J software to analyze scanned blots.

***Statistical analysis***

All values were presented as mean ± standard deviation (SD). For statistical analysis, the GraphPad Prism 8.0 Software for Windows (GraphPad Software Inc., La Jolla, CA, USA) was utilized. A one-way analysis of variance (ANOVA) was used among multiple groups, followed by Tukey’s multiple comparisons test. Statistical significance was defined as a *P*-value of 0.05.

## Results

***Andro improves histopathological injury in the lungs***

**Figure 1:**
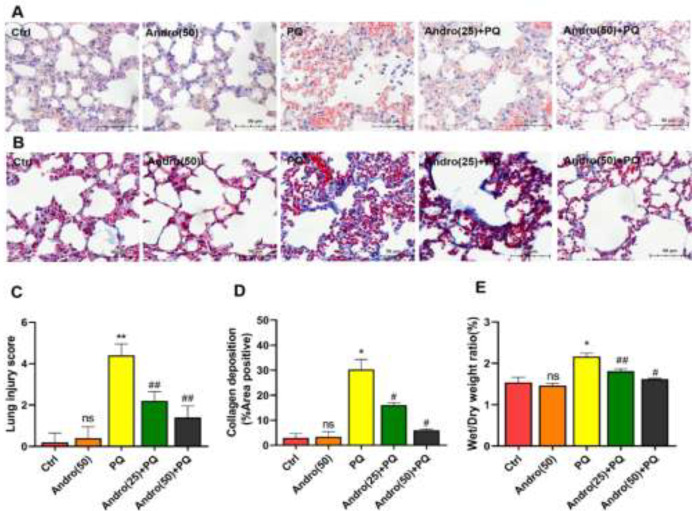
Andro alleviates PQ-induced lung injury in mice

In comparison with the Control and Andro alone groups, histological damage in the lungs following PQ exposure demonstrated alveolar structural deterioration, alveolar congestion, inflammatory cell infiltration, and a rise in collagen fiber deposition, as illustrated in Figure 1. Andro alleviated the PQ-induced lung histopathological injury, particularly at the higher dose of Andro (50  mg/kg). No statistical difference was shown in Control and Andro alone groups (*P*>0.05). Histological scores with PQ exposure were clearly the highest of any other group. However, Andro at various dosages dramatically reduced lung damage scores and collagen deposition caused by PQ. (*P*<0.01 for lung injury score and *P*<0.05 for collagen deposition) (Figure 1).

In this investigation, the ratio of W/D weight was assessed to validate Andro’s protective effects against pulmonary edema caused by PQ. The ratio of W/D weight in lungs caused by PQ was greater than those in the Control and Andro alone groups but was significantly lowered by Andro at 25 mg/kg and 50 mg/kg. (*P*<0.05 to *P*<0.01)(Figure 1).

***Andro inhibits PQ-induced inflammatory factors and oxidative stress in mice***

**Figure 2:**
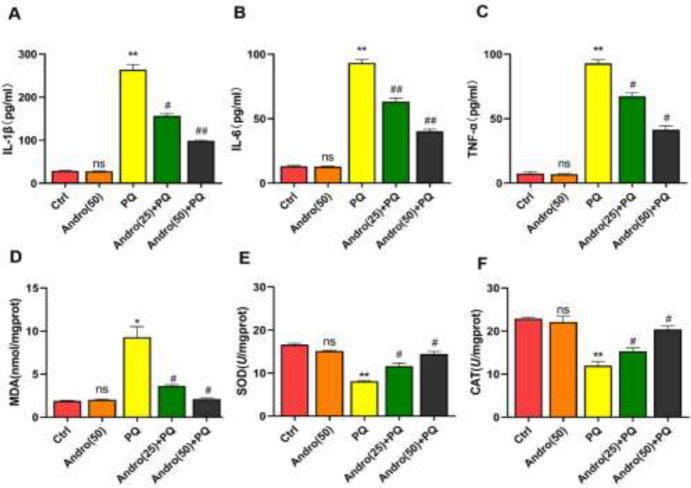
Andro inhibited PQ-induced inflammation and oxidative stress in the lungs

It was reported that there was clear lung edema and inflammatory cell infiltration in mice treated with PQ for 72 hr (30). Therefore, the protective benefits, and the possible mechanism of Andro on the lungs, treated with PQ, were studied: compared with control groups, PQ exhibited dramatic increases in IL-1β, IL-6, and TNF-α levels. But, Andro treatment dramatically reduced the increase of IL-1β, IL-6, and TNF-α levels caused by PQ in the lungs ( *P*<0.05 to *P*<0.01)(Figure 2).

Moreover, compared with Control and Andro alone groups, PQ exposure resulted in a noticeable increase in MDA expression, and a considerable drop in SOD and CAT levels. However, Andro significantly alleviated the increase in MDA levels induced by PQ, and elevated SOD and CAT levels in a concentration-dependent manner (*P*<0.05 to *P*<0.01)(Figure 2).

***Andro suppresses PQ-induced apoptosis in mice***

**Figure 3:**
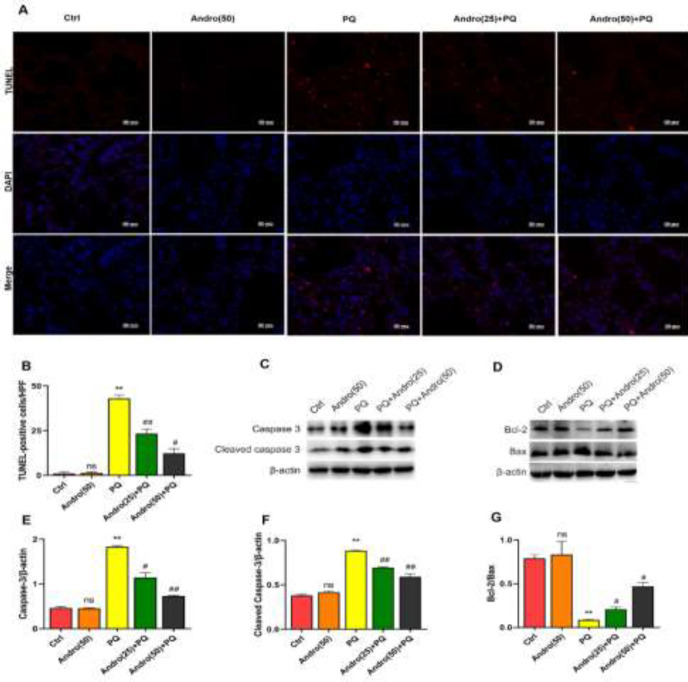
Andro decreased PQ-induced lung tissue apoptosis in mice

Lung tissue apoptosis was evaluated via TUNEL staining. Compared with Control and Andro alone groups, PQ exhibited a dramatically higher number of TUNEL-positive cells, as demonstrated in Figure 3A. However, Andro with different dose treatments significantly attenuated the PQ-induced increase of the apoptotic cells (*P*<0.05 to *P*<0.01)(Figure 3). 

To further evaluate the possible mechanism of apoptosis, Bcl-2/Bax ratio and proteins caspase-3 and cleaved caspase-3 were adopted. PQ revealed a substantial drop in the ratio of Bcl-2/Bax and an increase in caspase-3 and cleaved caspase-3 expression compared with the Control and Andro alone groups. Meanwhile, Andro administration effectively mitigated the PQ-induced decrease in the ratio of Bcl-2/Bax as well as the rise in caspase-3 and cleaved caspase-3 levels (*P*<0.05 to *P*<0.01) (Figure 3).

***Andro promotes Nrf2/HO-1 pathway activation in the lungs***

**Figure 4:**
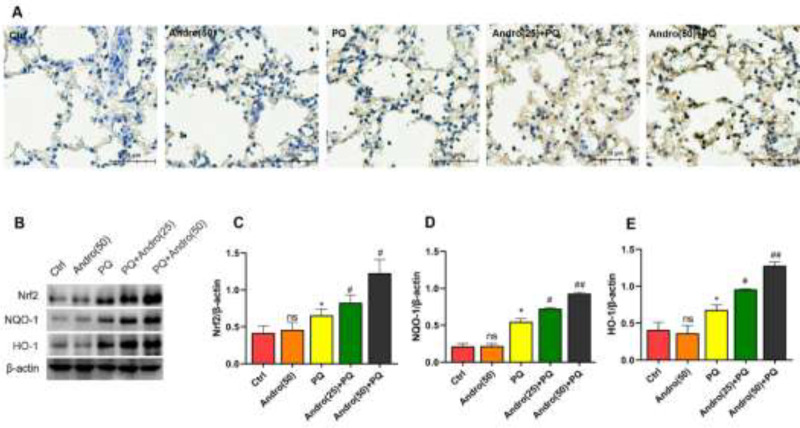
Effects of Andro on Nrf2/HO-1 in PQ-induced acute lung injury

The Nrf2 level of the lungs was assessed through IHC and Western blotting. PQ exhibited a dramatic increase in Nrf2 level compared with the Control and Andro alone groups. Andro significantly enhanced the level of Nrf2 by IHC and Western blot. Besides that, PQ treatment increased Nrf2-related pathway protein levels such as NQO-1 and HO-1. Moreover, Andro with 25 mg/kg and 50 mg/kg treatment significantly enhanced the PQ-induced increases of NQO-1 and HO-1 levels in the lungs. These findings, therefore, showed that PQ promoted the Nrf2/HO-1 pathway to become active during the disease’s early stages, while Andro significantly enhanced the Nrf2/HO-1 pathway (*P*<0.05 to *P*<0.01) (Figure 4).

***Andro reduces the inhibitory effects of PQ on MLE-12 cell viability***

**Figure 5:**
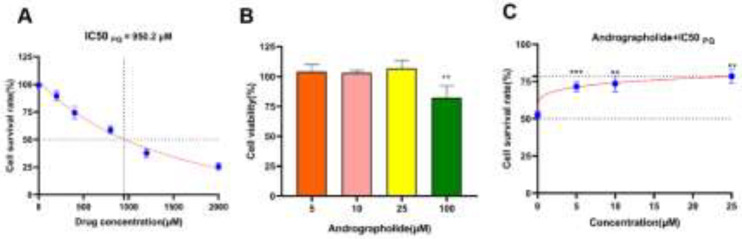
Andro facilitates the viability of MLE-12 cells

The optimal PQ and Andro concentration was determined by CCK-8 for the succeeding studies. All of these studies illustrated that PQ reduced cell viability in a concentration-dependent manner, with PQ at 950.2 M inhibiting cell viability by half. Thus, PQ with a concentration of 950.2 µM was chosen for a vitro model. Similarly, the cell viability of Andro on MLE-12 cells as well as the optimal concentration of Andro for reducing PQ-induced cytotoxicity were also determined. Notably, Results described that Andro alone had no cytotoxicity on MLE-12 cells until 100 µM, and Andro at 25 µM was optimal in reducing PQ cytotoxicity. Thus, the optimal dosage of Andro at 25 M was determined for the subsequent studies (*P*<0.01 to *P*<0.001)(Figure 5).

***Nrf2 knockout abolished the effects of Andro in inhibiting oxidative injury and apoptosis***

**Figure 6:**
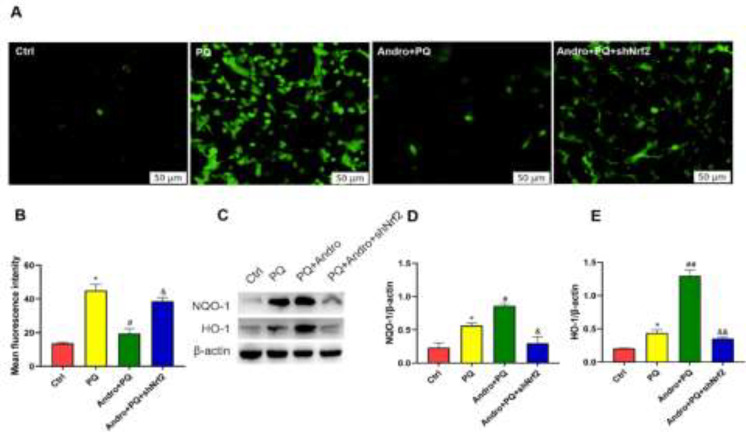
Mitigated protection of Andro following the enforced silence of Nrf2 in oxidative stress injury induced by PQ in MLE-12 cells

**Figure 7:**
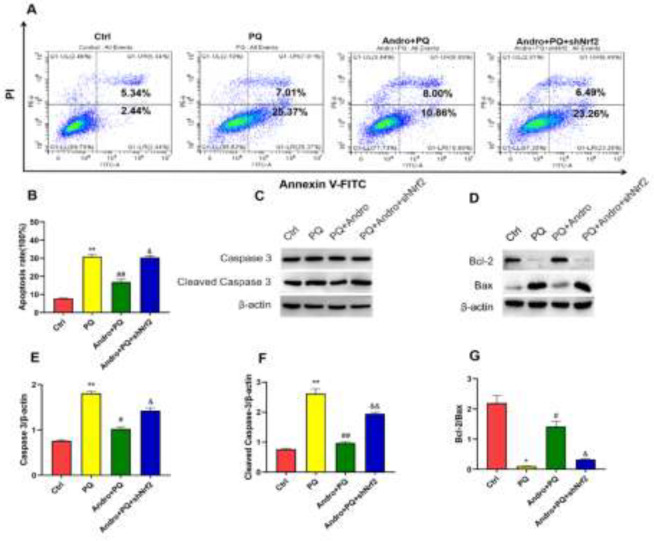
Andro attenuates the apoptosis induced by PQ by activating the Nrf2/HO-1 pathway in MLE-12 cells

As described in Figures 6 and 7, ROS and apoptosis in the MLE-12 cells significantly increased after PQ treatment but were reduced after pretreatment with Andro. However, ROS generation and apoptosis were reversed with Nrf2 knockout compared with the Andro treatment. Our findings demonstrated that inhibiting Nrf2 reduced Andro’s protective properties against ROS generation and apoptosis caused by PQ (*P*<0.05)(Figures 6 and 7). 

PQ exhibited increases in NQO-1 and HO-1 compared with the Control group. Also, pretreatment with Andro clearly enhanced NQO-1 and HO-1 levels after PQ exposure. The NQO-1 and HO-1 levels significantly decreased after Nrf2 was knocked out compared with Andro pretreatment (*P*<0.05 to *P*<0.01) (Figure 6).

Furthermore, to explore the possible mechanism by which Andro inhibits apoptosis, the ratio of Bcl-2/Bax, caspase-3, and cleaved caspase-3 were further investigated. Our findings showed that PQ exhibited significant increases in caspase-3 and cleaved caspase-3, but a significant decrease in Bcl-2/Bax compared with the Control group. However, Andro significantly improved the ratio of Bcl-2/Bax as well as the decrease of caspase-3 and cleaved caspase-3. However, the ratio of Bcl-2/Bax dramatically decreased, and the caspase-3 and cleaved caspase-3 levels markedly increased after Nrf2 was knocked out, compared with Andro treatment. These results, as described above, indicate that Andro exerts a beneficial effect on PQ-induced oxidant injury and apoptosis by promoting Nrf2/HO-1 pathway activation (*P*<0.05 to *P*<0.01)(Figure 7).

(A) Lung tissue injury assessment by H&E staining (scale bar = 50 μm). (B) Collagen deposition assessment by Masson trichrome staining (scale bar = 50 μm). (C) Lung injury score evaluation. (D) Collagen deposition area ratio. (E) Lung Wet/Dry weight ratio. Data are presented as the means ± SD (n=3 in each group). “ns” means no significant difference with the Control group;*P< 0.05, **P< 0.01 vs the Control group; #*P*<0.05,##*P*<0.01 vs the PQ group. Andro, Andrographolide; PQ, Paraquat. Comparisons between different groups were using one-way ANOVA followed by Tukey’s multiple comparisons test

(A) IL-1β, (B) IL-6, and (C) TNF-α as major inflammatory indicators were determined by Enzyme-linked Immunosorbent Assay (ELISA) in lung tissues. Markers of oxidative stress including (D) MDA content, (E) SOD activity, and (F) CAT content in the lungs were detected through the assay kits. The results are expressed as the means ± SD (n=3 in each group). “ns” means no significant difference with the Control group; *P<0.05, **P<0.01 vs the Control group; #*P*< 0.05,##*P*< 0.01 vs the PQ group. Andro, Andrographolide; PQ, Paraquat. Comparisons between different groups were using one-way ANOVA followed by Tukey’s multiple comparisons test

(A) Representative images of lung tissue apoptosis (TUNEL staining, scale bar = 50 μm). (B) Semi-quantitative analysis of lung tissue apoptosis. (C) Representative western blot images of caspase-3 and cleaved caspase-3. (D) Representative western blot images of Bcl-2 and Bax. Semi-quantitative analysis of (E) caspase-3, (F) cleaved caspase-3, and (G) the ratio of Bcl-2/Bax. The results are expressed as mean ± SD (n=3 in each group).“ns” means no significant difference with the Control group; *P<0.05, **P<0.01 vs the Control group; #*P*<0.05, ##*P*<0.01 vs the PQ group. Andro, Andrographolide; PQ, Paraquat. Comparisons between different groups were using one-way ANOVA followed by Tukey’s multiple comparisons test

(A) Immunohistochemical (IHC) assay was used to evaluate the expression of Nrf2 in the lungs (IHC, scale bar = 50 μm). (B) Western blotting analysis was performed to detect the expression of Nrf2, NQO-1, and HO-1 in the lungs in mice. Representative western blotting images and quantitative analysis of (C) Nrf2, (D) NQO-1, and (E) HO-1. The results are expressed as mean ± SD (n=3 in each group). “ns” means no significant difference with the Control group; *P< 0.05, **P< 0.01 vs the Control group; #*P*< 0.05, ##*P*< 0.01 vs the PQ group. Andro, Andrographolide; PQ, Paraquat. Comparisons between different groups were using one-way ANOVA followed by Tukey’s multiple comparisons test

(A) MLE-12 cell viability was suppressed when cells were treated with various PQ concentrations. (B) Effects of different Andro concentrations on the activity of MLE-12 cells. (C) Andro improved MLE-12 cell viability treatment with PQ. Data are presented as the means ± SD (n=3 in each group). Andro was added 6 hr prior to PQ treatment for 24 hr. **P< 0.01, ***P< 0.01 vs the Control group; Andro, Andrographolide; PQ, Paraquat. Comparisons between

different groups were using one-way ANOVA followed by Tukey’s multiple comparisons test

(A) The fluorescent probe, DCFH-DA, was used to assess the ROS expression levels in the forced silence of Nrf2 in MLE-12 cells. (B) Histograms of DCF fluorescence in each group. (C) Western blot analysis was performed to determine the protein expression of the Nrf2/HO-1 pathway in the enforced silence of Nrf2 in MLE-12 cells. Representative western blot images and quantitative analysis of (D) NQO-1 and (E) HO-1. Data are presented as the means ± SD (n=3 in each group). Andro was added 6 hr prior to PQ treatment for 24 hr. *P< 0.05, **P< 0.01 vs the Control group; #*P*< 0.05, ##*P*< 0.01 vs the PQ group. &*P*< 0.05, &&*P*< 0.01 vs the Andro+PQ group. Andro, Andrographolide; PQ, Paraquat. Comparisons between different groups were using one-way ANOVA followed by Tukey’s multiple comparisons test

(A) Andro significantly reduced the apoptosis induced by PQ and detected by flow cytometry. (B) Comparisons of apoptosis ratio in MLE-12 cells. (C) Western blotting analysis was performed to determine the protein expression of caspase-3 and cleaved caspase-3 in the enforced silence of Nrf2 in MLE-12 cells. (D) Western blotting analysis was performed to determine the protein expression of Bcl-2 and Bax in the enforced silence of Nrf2 in MLE-12 cells. Representative Western blot images and quantitative analysis of (E) caspase-3, (F) cleaved caspase-3, and (G) the Bcl-2/Bax ratio. Data are presented as the means ± SD (n=3 in each group). Andro was added 6 hr prior to PQ treatment for 24 hr. *P< 0.05, **P< 0.01 vs the Control group; #*P*< 0.05, ##*P*< 0.01 vs the PQ group. &*P*< 0.05, &&*P*< 0.01 vs the Andro+PQ group. Andro, Andrographolide; PQ, Paraquat. Comparisons between different groups were using one-way ANOVA followed by Tukey’s multiple comparisons test

## Discussion

PQ-induced pulmonary toxicity is studied because it is extremely susceptible to polyamine systems’ ingestion of the lungs, and it often gives rise to a variety of pathological damage resulting in ALI, initially, and irreversible pulmonary fibrosis ultimately (9). The mortality rate of PQ-induced poisonousness reaches an impressive 40%–90% due to accidental and/or international intake on account of no specific antidotes and effective therapeutic methods (31). However, some Chinese herbal extracts, such as ligustrazine and ginkgolide C, exhibit certain benefits and might be an alternative choice in reducing PQ-related disease processes (32, 33). Andro, mainly extracted from the *A. paniculata* herb, has many application values in resolving clinical problems owing to its significant antiviral, anti-inflammatory, and anti-oxidant activities (25, 26). However, whether Andro could alleviate PQ-mediated ALI remains unknown. As a result, the roles of Andro in relieving PQ-induced ALI, as well as the potential signaling pathways implicated in the process, were first probed in this research.

Previous research findings support the point that PQ-induced ALI is mainly associated with histopathological changes in the lungs, including alveolar structural damage, extent of leukocyte infiltration, pulmonary interstitial edema, collagen deposition, and alveolar hemorrhage (34-36). At present, results verify that a series of pulmonary pathological changes in PQ-induced ALI are in agreement with what we described above, which further supports our previous studies. However, Andro, in models of PQ-induced ALI, markedly alleviates PQ-induced pathological changes, including holding the structural integrity of alveoli, reducing severe leukocyte infiltration, relieving congestion and hemorrhage, and improving collagen deposition in the lungs. In addition, the increase of the ratio of W/D weight as a key indicator of the severity of pulmonary edema and the pro-inflammatory factor levels of IL-1β, IL-6, and TNF-α caused by PQ was reverted back by Andro. These significant changes can partially be attributed to the fact that Andro elicits a strong protective role in reducing ALI caused by PQ.

Oxidative injury is a well-recognized critical underlying mechanism in PQ-induced lung toxicity, and ROS is the main initiating factor of oxidative stress injury (37). Oxidative stress, induced by PQ, occurs when the anti-oxidant defense mechanism is overwhelmed by ROS, which is further confirmed by an apparent increase of ROS in PQ-induced MLE-12 cells. ROS promotes lipid peroxidation, destabilizes the cell membrane, and produces reactive aldehyde byproducts such as MDA, which actively participates in ALI by activating inflammatory responses and the occurrence of apoptosis (38, 39). Meanwhile, anti-oxidant levels may increase in potent enzymes, such as SOD and CAT anti-oxidants, to eliminate the harmful effects of ROS after PQ exposure (40). Furthermore, our findings demonstrated significantly elevated levels of ROS and MDA following PQ exposure in mice and MLE-12 cells, supporting the important role of oxidant stress in lung toxicity caused by PQ (41). Meanwhile, several anti-oxidants, such as SOD and CAT, decreased in mice, which might be attributed to the increased consumption in response to MDA and ROS overproduction once the anti-oxidant defense mechanism was turned on. Therefore, oxidative stress caused by PQ mainly is attributed to the oxidation and anti-oxidant imbalance by an increase in the amounts of ROS and MDA. Andro, however, prominently improves the anti-oxidant capability by promoting the synthesis of SOD and CAT and eliminating ROS and MDA, which further confirmed the anti-oxidant capability of Andro in reducing PQ-induced ALI.

More interestingly, accumulating evidence has illustrated that the Nrf2/HO-1 signaling pathway, as an essential pathway regulating the synthesis of anti-oxidant enzymes, plays a critical role in mitigating PQ-induced oxidative injury (42-44). Our experiment results also support the point that the anti-oxidant transcription factor of Nrf2 is raised in PQ-induced ALI in mice and MLE-12 cells. Nrf2 attaches to Kelch-like ECH-associated protein 1 (Keap 1) and exists in an inactive state in the cytoplasm. Once oxidative stress induced by PQ is triggered, Nrf2 immediately dissociates from Keap 1, translocates into the nucleus, binds to the anti-oxidant response element (ARE), and induces the expression of phase-II detoxing enzymes, including NQO-1 and HO-1, to promote the generation of the anti-oxidant proteins, such as SOD and CAT, to initiate the anti-oxidative defense to avert oxidative stress injury (20). Also, our findings indicated that the initial stage of PQ-induced lung toxicity manifested the increase of Nrf2, NQO-1, and HO-1, in agreement with previous studies, suggesting that the Nrf2/HO-1 anti-oxidative pathway was initiated at the beginning in an attempt to restore the oxidation-anti-oxidant balance and maintain the stability of cell structure and function (45). Furthermore, the Nrf2/HO-1 anti-oxidant pathway was markedly activated by Andro in a concentration-dependent manner in mice after PQ exposure. However, the key anti-oxidant protein levels of the Nrf2/HO-1 pathway were remarkably decreased with Nrf2 knockout *in vitro*, as well as excessive ROS production. All these findings demonstrated that Andro triggered the Nrf2/HO-1 pathway in order to reduce PQ-mediated oxidative injury.

In addition, apoptosis has been identified as a crucial factor in PQ-induced ALI (9, 46). Apoptosis is mediated by two well-known signaling mechanisms, the extrinsic and intrinsic pathways. The extrinsic pathway is activated by external death ligands; the intrinsic pathway is triggered by internal apoptotic signals and is involved in the mitochondria. The two pathways merge and share mechanisms utilizing the aspartate-specific cysteinyl protease (caspase) cascades. Furthermore, caspase-3 is an important central effector that initiates apoptosis and cleaves caspase-3, which is responsible for the main execution of apoptosis. The intrinsic pathway involves Bcl-2 and Bax, which respectively represent the Bcl-2 family as anti-apoptotic and pro-apoptotic molecules that regulate apoptosis (47, 48). Our findings revealed that PQ induced apoptosis in mice and MLE-12 cells, and was involved in its underlying mechanism by increasing caspase-3, cleaving caspase-3, and decreasing the ratio of Bcl-2/Bax. This indicated that apoptosis was closely associated with PQ-induced ALI through the intrinsic pathway. However, Andro significantly ameliorated PQ-induced apoptosis by inhibiting caspase-3 and cleaved caspase-3, and by promoting the ratio of Bcl-2/Bax. Moreover, to further explore the possible underlying mechanism of Andro in inhibiting apoptosis, the gene Nrf2 was knocked out by cell transfection. As we expected, the incidence of apoptosis events remained high even with Andro intervention. This phenomenon is related to the changes in anti-apoptotic proteins and pro-apoptotic proteins. In this study, the pro-apoptotic levels of caspase-3 and cleaved caspase-3 were distinctly elevated, and the Bcl-2/Bax ratio dramatically declined after the gene Nrf2 was knocked out, which further indicates that Andro induces the activation of the Nrf2/HO-1 signaling pathway to promote the generation of Bcl-2 and reduce the levels of Bax, caspase-3 and cleaved caspase-3 in order to inhibit apoptosis. Therefore, these results, being reported for the first time, indicate that Andro promotes Nrf2/HO-1 signaling pathway activation in order to ameliorate PQ-mediated apoptosis.

## Conclusion

In summary, our findings indicated that Andro effectively protected against PQ-induced ALI, mainly by reducing oxidative injury and inhibiting apoptosis *in vivo *and* in vitro*. These protective effects are primarily attributed to the Nrf2/HO-1 signaling pathway activation, which inhibits apoptosis by lowering the pro-apoptotic proteins caspase-3 and cleaved caspase-3 levels, as well as by increasing the ratio of Bcl-2/Bax. Therefore, these findings open up new possibilities for the treatment of PQ-induced ALI.

## Authors’ Contributions

QY and DZ designed the experiments; FS, SM, JH, XC, SN, YM, LR, and HL performed experiments and collected data; DZ discussed the results and strategy; QY supervised, directed, and managed the study; DZ, FS, SM, JH, XC, SN, YM, LR, HL, and QY approved the final version to be published.

## Conflicts of Interest

None.
